# Inteins, introns, and homing endonucleases: recent revelations about the life cycle of parasitic genetic elements

**DOI:** 10.1186/1471-2148-6-94

**Published:** 2006-11-13

**Authors:** J Peter Gogarten, Elena Hilario

**Affiliations:** 1 Department of Molecular and Cell Biology, University of Connecticut, Storrs, Connecticut 06269-31258, USA; 2 HortResearch, 120 Mt. Albert Road, Private Bag 92 169, Mt. Albert, Auckland, New Zealand

## Abstract

Self splicing introns and inteins that rely on a homing endonuclease for propagation are parasitic genetic elements. Their life-cycle and evolutionary fate has been described through the homing cycle. According to this model the homing endonuclease is selected for function only during the spreading phase of the parasite. This phase ends when the parasitic element is fixed in the population. Upon fixation the homing endonuclease is no longer under selection, and its activity is lost through random processes. Recent analyses of these parasitic elements with functional homing endonucleases suggest that this model in its most simple form is not always applicable. Apparently, functioning homing endonuclease can persist over long evolutionary times in populations and species that are thought to be asexual or nearly asexual. Here we review these recent findings and discuss their implications. Reasons for the long-term persistence of a functional homing endonuclease include: More recombination (sexual and as a result of gene transfer) than previously assumed for these organisms; complex population structures that prevent the element from being fixed; a balance between active spreading of the homing endonuclease and a decrease in fitness caused by the parasite in the host organism; or a function of the homing endonuclease that increases the fitness of the host organism and results in purifying selection for the homing endonuclease activity, even after fixation in a local population. In the future, more detailed studies of the population dynamics of the activity and regulation of homing endonucleases are needed to decide between these possibilities, and to determine their relative contributions to the long term survival of parasitic genes within a population. Two outstanding publications on the amoeba *Naegleria *group I intron (Wikmark et al. *BMC Evol Biol *2006, **6:**39) and the PRP8 inteins in ascomycetes (Butler et al.*BMC Evol Biol *2006, **6:**42) provide important stepping stones towards integrated studies on how these parasitic elements evolve through time together with, or despite, their hosts.

## 

In the organism centered view of evolution, the individual is considered the unit of selection. By being fit, the individual's genes have a greater chance to become fixed in the population. Challenging this view, Dawkins introduced a gene centered view of evolution [1] where the individual organism is just a vessel created by selfish genes. Cooperative genes rely on the individual's success – they increase their frequency in the population through increasing the fitness of the organism; in contrast, the perpetuation of parasitic genes through generations is independent of the host's fitness. The molecular parasites have gained their own individuality, and to understand their evolution, one has to consider their life cycle. Frequently, these parasitic elements are labeled as selfish genes; however, according to Dawkins [1] all genes are selfish, thus we prefer the label "parasitic" to reflect the fact that these genes are not cooperating to the benefit of the host organism.

Self splicing introns and inteins are remarkable examples of parasitic elements [2-7]. These molecular parasites can excise themselves of the host molecule (mRNA, rRNA or tRNA for introns; protein for inteins) and ligate the ends of the host molecule without perturbing its biological function. Some of these molecular parasites are equipped with homing endonucleases (HE) [8-10], which produce a single double-strand cut in the genomic DNA, usually in the intein- or intron-free allele of the infected gene. During DNA repair, the parasitic element is copied into the previously empty allele. In sexual populations this invasion of uninfected alleles leads to super Mendelian inheritance of the molecular parasites. It is curious to note that the spread of these parasites in the population relies on the different alleles being brought together through sex or gene transfer; however, many mobile elements with HE activity are found in organisms and organelles thought to be nearly clonal (e.g., mitochondria and prokaryotes) or relying on asexual genetic exchange, e.g., fungi [11] and amoeba [12] without known sexual recombination.

The fitness of the molecular parasite and the fitness of the organismal host walk separate roads [8,13]. For the parasite, fitness is reflected in its ability to spread in the population. Because of its splicing activity the parasite does not impact on the host protein function. The parasite actively colonizes all individuals until the entire population contains the intron/intein. Selection acts upon the splicing properties at all times, but for the HE activity selection only operates during invasion. When the parasitic element is fixed in the population, the HE function decays and is eventually lost. Goddard and Burt [13] first formulated the homing cycle (Fig. 1) for introns with HE activity; a modified model was applied to inteins [3,14].

**Figure 1:**
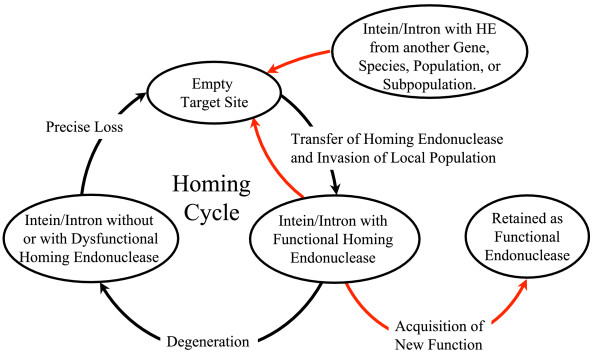
Homing cycle of a parasitic genetic element (modified from [3, 13]). Recent findings suggest that due to complex population structure the cycle might not operate in synchrony in different subpopulations. The red arrows indicate the trajectory of the functioning HE and the black arrows the fate of the host gene. The precise loss can occur through recombination with an intein or intron free allele, or, in case of introns, through recombination with a reverse transcript of the spliced mRNA [39, 40].

Conceptually, the homing cycle should be formulated for interbreeding populations [3], i.e., the units in which the parasitic genetic element can be fixed; however, often species, not populations, were considered as units in which the HE containing element was fixed, and the presence of a gene with active HE was interpreted as reflecting gene exchange across species boundaries. These assumptions were frequently confirmed by more detailed analysis of species, host protein, and HE phylogenies [13-16]. The presence of HE containing introns and inteins thus becomes an indicator for DNA exchange events within or across the species boundaries, for example, transfers between mitochondrial genomes in plants and fungi [13-16], or from eukaryotes to extreme thermophilic bacteria [17].

## Recently discovered limitations of the homing cycle model

Several recent findings challenge the general applicability of the homing cycle: in *Naegleria*, an apparently asexual amoeba, an intron with HE was reported to be of ancient origin, frequently lost in different lineages, but persisting as a functional enzyme in others [18]. And in three different orders of euascomycetes (Pezizomycota) inteins were discovered in the PRP8 gene [19], all inserted at the same location within the gene. The PRP8 intein has been horizontally transferred between euascomycetes and the basidiomycete *Cryptococcus *[19] but no evidence for transfer between the different lineages of euascomycetes was detected. Some of the PRP8 inteins in euascomycetes contain a functional HE under purifying selection, as judged by the ratio of synonymous to non-synonymous substitutions [19]. According to a recent attempt to date fungal evolution [20], the groups of euascomycetes that contain a PRP8 intein already diverged in the Late Proterozoic (i.e., before 540 million years BP). Could a functioning HE survive within a species over several hundred million years without interspecies transfer?

We discuss several possible explanations, not all complementary, for these findings:

(A) Sex and genetic exchange occur more frequently than assumed. Rare events of genetic exchange that bring HE containing alleles together with intron/intein free alleles might be sufficient to maintain the HE under purifying selection.

(B) The HE might have a function beneficial to the host. In case of *Bacillus *phages, introns with HE were reported to increase the fitness of their respective host by destroying the genomes of competing phages, containing a different HE, that co-infected the same host [21]. However, selection at the gene and the population level are interwoven tightly in this case and an alternative explanation considering only the gene's selfishness and competition of different HEs for target sites was proposed [22]. Many self splicing introns encode maturases that assist in the splicing reaction catalyzed by the intron [23], and some of these evolved from homing endonucleases [24]. Again selection at the different levels is interwoven, the intron now relies on the HE not only for spreading within the population, it also requires the HE for splicing. Without the maturase activity, the intron splices less effectively and thus the absence of the maturase/HE is detrimental to the host. The maturase is under purifying selection, as long as the intron is present. This sequence of events illustrates a neutral pathway to complexity [25]: The organism harboring the self-splicing intron is no better off than without the intron, but it now requires a more complex machinery to catalyze the splicing reaction, and the parts required for splicing now are under purifying selection. However, as long as the maturase/HE only acts on the encoding intron, the homing cycle could continue with the simultaneous deletion of the intron and the encoded maturase/HE. In *Saccharomyces cerevisiae *an endonuclease that triggers recombination events leading to mating type changes is a homolog of the HE containing intein in the vacuolar ATPase catalytic subunit [3,26,27]; and a genome rearrangement function was also suggested in *Thermococcus kodakaraensis *[28]. The acquisition of a new function that allowed the HE to exit from the homing cycle also occurred in plants and in soft corals where a mitochondrial mutS homolog fused twice independently with different HE types [29].

(C) The homing endonuclease might be maintained by balancing selection. Butler et al [19] suggested that the intein might decrease the fitness of the host organism. The long term survival of the functioning HE might result from the active invasion due to conversion of intein free alleles, balanced by the decreased propagation of the infected hosts. This balance could be achieved without the homing cycle operating in the population.

(D) The homing cycle might operate in subpopulations only. A low homing frequency might be sufficient to provide purifying selection for the HE function, but insufficient to fix the parasite in the whole population simultaneously. The process of fixation might be further delayed by a complex population structure. Decay and loss of the parasites' fitness already might occur in one subpopulation, whereas other subpopulations might be only at the beginning of the invasion phase. The resulting situation is comparable to waves in an excitable medium that run in circles, as in a fibrillating heart-muscle (re-entry ventricular fibrillation[30,31]). In the spatial model the homing cycle operates on smaller sections of the population; the population or the species as a whole would be out of synchrony. Reinvasion therefore can occur from within the species or population.

Hypothesis A is compatible with the other three scenarios, and the presence of a HE containing parasitic gene already suggests a low level of gene flow occurring in these presumably clonal organisms. It will be interesting to learn how the homing process in these organisms begins – in the best studied example, the *vma-1 *intein in *Saccharomyces cerevisiae *[32-34], homing only occurs during meiosis [35,36]. Hypothesis B might explain the persistence of a class I intron, if the encoded HE had acquired a maturase function [24] acting in trans on other self-splicing introns; however, under this assumption it remains puzzling that most *Naegleria *isolates lost the intron. The same argument applies to the PRP8 intein: The acquisition of a new function is at odds with the presence of several PRP8 genes in euascomycetes that lost either the HE domain or the whole intein altogether. If the HE acquired a function beneficial to the host, why was it lost repeatedly during evolution?

Hypothesis C is unlikely to be a sufficient explanation on its own. It would require an exact balance between forces that increase and those that decrease the HE frequency. A slight shift away from the equilibrium would mean either fixation or extinction for the HE containing parasite. However, an approximate balance between decreased fitness of the host and active spreading of the parasitic element, combined with a complex population structure could lead to long persistence times without the homing cycle operating, especially when the selective disadvantage to the carrier and the mobility of the element were not the same in different subpopulations. This hypothesis on the surface appears similar to hypothesis D; however, in the absence of the homing cycle the empty target sites would not result from decay and loss of the parasitic element, but from the faster growth rate of the organisms that never possessed the element.

The asynchronous homing cycle (hypothesis D) is a likely contributor to the long term persistence of functional HE in a single species. To corroborate this hypothesis, and to differentiate it from hypothesis C, more detailed population studies need to be performed, including sampling of isolated subpopulations with limited migration between them, and thorough sampling of well mixed isolated populations. In addition, a sampling of related species is also necessary since the detection of interspecies transfers depends on a sufficient sampling of taxa. At present it cannot be excluded that some interspecies transfer of the HE went undetected.

The unexpected complexity in the life cycle of inteins reported by Butler and colleagues reinforces the need for detailed epidemiological studies of HE containing parasitic genes. These studies should be combined with attempts to better understand the biochemical and physiological regulation of the parasitic genes, with measurements of their effect on the fitness of their host, with determination of the transmission efficiency of the parasite, and with phylogenetic analyses that might reveal the frequency of HE loss form the parasitic gene. In case of introns the study of the epidemiological dynamics can be further complicated by several factors (see [37] for a recent review): HE genes and self-splicing introns can be considered independent parasites and a HE can associate with different introns that provide integration sites that are selectively neutral; self-splicing introns are frequently found in ribosomal RNA encoding genes that are present in multiple copies per genome; in addition to using HEs, introns can also be mobile through reverse splicing; and the HE function might also assist the splicing reaction. The epidemiological dynamics of the HE containing parasitic genes are complex, and the determination of the relevant parameters will require collaborative efforts by molecular biologists, microbial ecologists, and epidemiologists. However, these studies will be worthwhile, because they provide a chance to untangle the interactions between the selective forces acting at the molecular and the organismal levels, and they also will allow detection of low rates of gene flow between populations and possibly between species. These studies thus will constitute important steps towards a more detailed genome 'ecology' [38].
